# Two series of new semisynthetic triterpene derivatives: differences in anti-malarial activity, cytotoxicity and mechanism of action

**DOI:** 10.1186/1475-2875-12-89

**Published:** 2013-03-09

**Authors:** Gloria NS da Silva, Nicole RG Maria, Desirée C Schuck, Laura N Cruz, Miriam S de Moraes, Myna Nakabashi, Cedric Graebin, Grace Gosmann, Célia RS Garcia, Simone CB Gnoatto

**Affiliations:** 1 Phytochemistry and Organic Synthesis Laboratory, School of Pharmacy, Federal University of Rio Grande do Sul, Porto Alegre, 90610-000, Brazil; 2 Plasmodium Molecular and Cellular Biology Laboratory, Department of Physiology, São Paulo University, São Paulo, 05508-900, Brazil; 3 Department of Chemistry, Federal Rural University of Rio de Janeiro, Seropedica, 23897/000, Brazil

**Keywords:** *Plasmodium falciparum*, Anti-malarial, Mitochondrial membrane potential, β-haematin, Betulinic acid, Ursolic acid, Semisynthesis

## Abstract

**Background:**

The discovery and development of anti-malarial compounds of plant origin and semisynthetic derivatives thereof, such as quinine (QN) and chloroquine (CQ), has highlighted the importance of these compounds in the treatment of malaria. Ursolic acid analogues bearing an acetyl group at C-3 have demonstrated significant anti-malarial activity. With this in mind, two new series of betulinic acid (BA) and ursolic acid (UA) derivatives with ester groups at C-3 were synthesized in an attempt to improve anti-malarial activity, reduce cytotoxicity, and search for new targets. *In vitro* activity against CQ-sensitive *Plasmodium falciparum* 3D7 and an evaluation of cytotoxicity in a mammalian cell line (HEK293T) are reported. Furthermore, two possible mechanisms of action of anti-malarial compounds have been evaluated: effects on mitochondrial membrane potential (Δ*Ψm*) and inhibition of β-haematin formation.

**Results:**

Among the 18 derivatives synthesized, those having shorter side chains were most effective against CQ-sensitive *P. falciparum* 3D7, and were non-cytotoxic. These derivatives were three to five times more active than BA and UA. A DiOC_6_(3) Δ*Ψm* assay showed that mitochondria are not involved in their mechanism of action. Inhibition of β-haematin formation by the active derivatives was weaker than with CQ. Compounds of the BA series were generally more active against *P. falciparum* 3D7 than those of the UA series.

**Conclusions:**

Three new anti-malarial prototypes were obtained from natural sources through an easy and relatively inexpensive synthesis. They represent an alternative for new lead compounds for anti-malarial chemotherapy.

## Background

Malaria is among the most significant of infectious diseases, influencing the health of humankind to this day [1]. *Plasmodium falciparum* is a protozoan parasite and the causative agent of the most virulent form of malaria in humans. In endemic areas, malaria accounts for nearly one million deaths, primarily among children under the age of five [2].

Higher plants are commonly used as sources for the discovery of new drug leads. Quinine (QN) and artemisinin are examples of plant-derived products with anti-malarial activity [3]. QN, one of the oldest and most important anti-malarial agents, has long been extracted from the bark of *Cinchona* (Rubiaceae) species [4]. Many other natural and semisynthetic anti-malarial compounds have since been developed, most of which fall into three classes: the quinolines, the artemisinin-based anti-malarials and the antifolates [5]. Nevertheless, due to the emergence of resistant *Plasmodium* strains, the therapeutic impact of these compounds has declined, urging development of new and effective anti-malarials [6]. The lack of low-cost and non-toxic drugs creates a very disturbing scenario for malaria management, and greater efforts must be made toward the discovery of new anti-malarial compounds [7]. The natural compound betulinic acid (3β-hydroxy-lup-20(29)-en-28-oic acid, BA) has been shown to exhibit a variety of biological activities, including inhibition of human immunodeficiency virus (HIV), antibacterial, anti-inflammatory, anticancer and anti-helminthic effects [8,9]. The *in vitro* anti-plasmodial activity (IC_50_) of BA against chloroquine (CQ)-resistant (K1) and CQ-sensitive (T9-96) *P. falciparum* strains was found to be 19.6 μg/mL and 25.9 μg/mL, respectively. In a murine malaria model using *Plasmodium berghei* NK65 strain, BA exhibited some toxicity when a high dosage was employed (250 mg/kg/day), but no toxicity was observed at a dose of 125 mg/kg/day [10].

**Figure 1:**
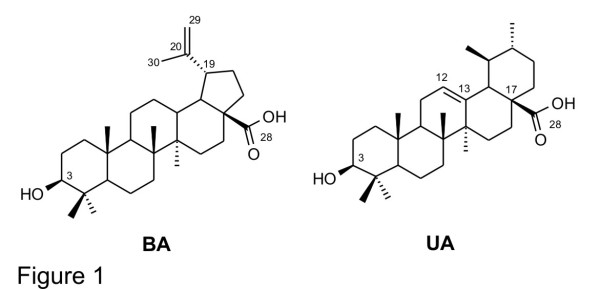
Betulinic (BA) and ursolic (UA) acids.

Another triterpene of pharmacological importance is ursolic acid (3β-hydroxyurs-12-en-28-oic acid, UA), which is known to have anti-inflammatory and antihyperlipidemic properties as well as antitumour-promoting effects [11]. Moreover, UA has been shown to suppress parasitaemia (96.9% at 60 mg/kg/day) in *P. berghei*-infected mice and reduce *in vitro* proliferation of *P. falciparum* strains 3D7, W2, and K1 [10,12]. The mechanism of action of triterpenes as anti-malarials is not fully understood. Gnoatto *et al.*[13] described seven new ursolic acid analogues bearing an acetyl group at C-3 and a piperazine moiety at C-28 with significant anti-malarial activity in the nanomolar range. The hydrophobic interactions of the UA skeleton were also characterized through molecular dynamic simulations, supporting the binding of triterpene to haem as a mechanism of action for these new UA derivatives. Therefore, the search for new triterpene derivatives is a promising approach for development of drugs with potential anti-malarial activity (Figure 1).

In this context, the present study report the synthesis and anti-malarial activity (against CQ-sensitive *P. falciparum* 3D7) of two new series of BA and UA analogues possessing various ester substituents at C-3, as well as the *in vitro* cytotoxicity of the most active compounds in HEK293T mammalian cells and their effect on mitochondrial membrane potential (*ΔΨm*) and inhibition of β-haematin formation*.* These mechanisms of action are likely involved in the anti-malarial activity of atovaquone and CQ respectively [14,15].

## Methods

### Collection of plant materials and isolation of BA and UA

*Platanus acerifolia* bark was collected in Porto Alegre, RS, Brazil. After botanical identification, a voucher specimen, ICN 171329, was deposited in the Herbarium of the Federal University of Rio Grande do Sul Department of Botany. BA was isolated as described in the additional material (Additional file 1) and its structure was confirmed using spectroscopic data and comparison with the existing literature [16]. UA was isolated from waste apple (*Malus domestica*) peels obtained from a local juice factory. This compound was identified using full spectroscopy data, which consistent with those previously described [13].

### Semisynthesis of the BA and UA series

**Figure 2:**
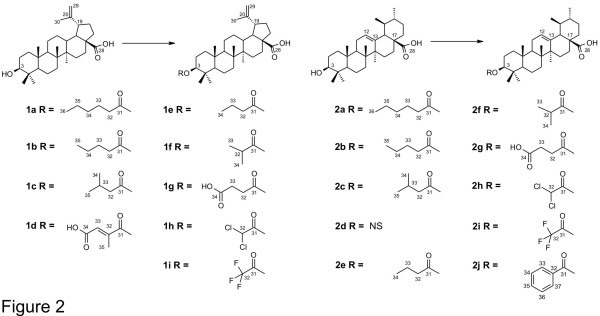
**Semisynthetic derivatives of series BA and UA.** NS: Not synthesized.

All commercially available reagents were used without further purification unless otherwise stated. Solvents were distilled under a positive-pressure dry nitrogen atmosphere when necessary. Reactions requiring anhydrous conditions were performed under a nitrogen atmosphere. BA and UA derivatives were synthesized according to the general procedure described in the literature [17]. Briefly, the appropriate anhydride (1.1 mmol, 5 Eq) and DMAP (0.22 mmol, 1 Eq) were added to BA or UA (0.22 mmol) in pyridine or CH_2_Cl_2_ (2 mL) and refluxed for 24 hours (for cyclic anhydrides) or processed without refluxing for one hour (for acyclic anhydrides). Column chromatography of the crude residue was performed to give the expected pure compounds. Series BA and UA are illustrated in Figure 2.

Column chromatography was carried out using silica gel 60 (Merck). Analytical thin layer chromatography was performed on silica gel 60 F_254_ plates (Merck) and spots visualized by spraying with anisaldehyde/sulphuric acid solution followed by heating at 100°C. Melting points were determined using a Kofler bench. High-resolution mass spectra (HR-EI-MS) were obtained on a Micromass-Waters Q-TOF Ultima spectrometer. Infrared spectra were recorded on a PerkinElmer FTIR BX spectrometer. ^1^H and ^13^C Nuclear Magnetic Resonance (NMR) spectra were recorded on a Bruker AC 400 spectrometer operating at 300 and 75 MHz, respectively, using tetramethylsilane as the internal standard and chloroform-d. The chemical shifts (δ) were expressed in parts per million (ppm).

### Determination of *in vitro* anti-malarial activity

*Plasmodium falciparum* (3D7 strain) was cultured in RPMI 1640 supplemented with 37.5 mM HEPES, 7 mM D-glucose, 6 mM NaOH, 25 μg/mL gentamicin sulfate, 2 mM L-glutamine and 10% human serum and maintained in human erythrocytes under a gas mixture of 5% O_2_, 5% CO_2_, and 90% N_2_[18]. Culture was synchronized by using 10% sorbitol [19]. Infected red blood cells (iRBC) with a parasitaemia of 1–2% and a haematocrit of 2% were incubated with compounds over a concentration range of 0.001 to 100 μM for 48 hours. CQ was included as positive control and the negative control was the solvent. Stock solutions of CQ and test compounds, prepared in water and dimethylsulfoxide (DMSO) respectively, were serially diluted with culture medium and added to synchronous parasite cultures in 96-well plates, following the addition of 200 μL formaldehyde solution 2% per well, overnight. Parasitaemia was evaluated by Giemsa-stained smears and flow cytometry. iRBCs were stained with YOYO®-1. The concentration required to inhibit parasite growth was determined by comparing the fluorescence of the treated and untreated (control) cultures. The concentration causing 50% inhibition (IC_50_) was obtained from the drug concentration response curve. The DMSO concentration never exceeded 0.1% and did not inhibit parasite growth. The definition of anti-malarial activity used herein was: IC_50_ ≤1 μM, potent activity; IC_50_ 1-10 μM, good activity; IC_50_ 10-30 μM, moderate activity; IC_50_ ≥30 μM, inactive [20]. Reagents were purchased from Merck and Sigma-Aldrich.

### *In vitro* cytotoxicity

Cytotoxicity was estimated using HEK293T (human embryonic kidney) cells. This cell line was cultured in 75 sq cm vented tissue culture flasks at 37°C in a humidified atmosphere containing 5% CO_2_, in Dulbecco’s modified essential medium (Gibco BRL) supplemented with 10% (v/v) foetal bovine serum, 100 U/mL penicillin, and 100 μg/mL streptomycin. Cells (5.0 x 10^4^/well) were seeded into 48-well plates. Twenty-four hours after plating, cells were treated with decreasing concentrations (100; 10; 1; 0.1; 0.01 and 0.001 μM) of compounds **1e**, **1f** and **2e** and evaluated after 24 and 48 hours. After incubation, cells were harvested with trypsin, gently centrifuged and resuspended in phosphate buffer saline. The cells were stained with dihydroethidium solution (10 mg/mL in phosphate buffered saline), gently vortexed, and incubated for 40 min at 37°C in the dark. Ten thousand gated events were acquired for each sample (FACSCalibur, Becton & Dickinson, and FlowJo software). The percentage of cell viability calculated as intrinsic cytotoxicity is represented by the concentration leading to 50% of cell death (IC_50_) on flow cytometry.

### Determination of changes in mitochondrial membrane potential (*ΔΨm*)

Changes in mitochondrial membrane potential were assessed as per Srivastava *et al.*[14]. Accumulation of the lipophilic cationic fluorescent probe 3,3^′^-dihexyloxacarbocyanine iodide (DiOC_6_(3)) in mitochondria was assessed in the presence of the active compounds **1e**, **1f** and **2e** and in their absence, in comparison with cells treated with the mitochondrial respiratory chain uncoupler carbonyl cyanide *m*-chlorophenyl hydrazone (CCCP). In this manner, iRBC in RPMI 1640 medium containing 10% human serum and 10% parasitaemia were incubated with a 2 nM final concentration of DiOC_6_(3) for 30 min at 37°C. Various concentrations of the compounds were then added to the culture and incubated for an additional 30 min at 37°C. At the end of the incubation period, samples were centrifuged for 5 min at 3,700 rpm and the supernatant discarded. Samples were then resuspended in phosphate buffer saline and 10^5^ events analyzed by flow cytometry.

### Inhibition of β-haematin formation

Inhibition of β-haematin formation was determined as described by Baelmens *et al.*[21]. CQ was used as the positive control. Briefly, 100 μL of a fresh 6.5 mM solution of haemin in 0.2 M NaOH was mixed with 200 μL of 3 M sodium acetate, 25 μL of 17.4 M acetic acid and 25 μL of the tested compound or the solvent as negative control (DMSO for **1e**, **1f** and **2e** or water for CQ). The final concentration of compounds in wells ranged from 0.5 to 20 mM. After 24 hours of incubation while gently shaking at 37°C, samples were centrifuged for 15 min at 3,300 *g*, the supernatant discarded and the pellet washed with 200 μL DMSO. This latter step was repeated once more and the pellet was finally washed with water. The pellet was then dissolved in 200 μL of 0.1 M NaOH. After a further 1:8 dilution, absorption at 405 nm was measured using a FlexStation plate reader. Experiments were carried out at least in triplicate. Results are expressed as percentage of inhibition of β-haematin formation in comparison to the negative control result, as calculated with the equation

$$\%\;\mathrm{Inhibition}=100*\left(1-\left(\mathrm{OD}\;\mathrm{drug}\right)/\left(\mathrm{OD}\;\mathrm{solvent}\right)\right)$$

### Statistical analysis

All results are expressed as mean ± standard error or mean ± standard deviation of at least three individual experiments. Student’s *t* test was chosen for between-group comparisons, whereas repeated measures ANOVA was used for comparisons amongst more than two groups. Statistical significance was defined as *p <* 0.05. Analyses were carried out in GraphPad Prism version 4.00 for Windows (GraphPad Software, San Diego, CA USA).

## Results

### Description of natural triterpenes and their synthetic derivatives

The structures of the isolated compounds are shown in Figure 1, and their synthetic derivatives are shown in Figure 2.

The triterpene BA was isolated from *P. acerifolia* bark, and UA from *M. domestica* peel; both sources were highly advantageous due to their low cost and good yields (2% for BA and 2.8% for UA; expressed in % weight/weight of dried bark or raw material). After semisynthesis of the eighteen compounds in series BA and UA, their structural elucidation could be deduced from IR, ^1^H, ^13^C NMR, HR-EI-MS and elemental analysis data (Additional file 2).

### *Plasmodium falciparum* susceptibility

Table 1 presents the IC_50_ for the isolated and synthesized compounds against CQ-sensitive *P. falciparum* 3D7 (IC_50_CQ = 29 nM). The IC_50_ for BA and UA were 18 and 36 μM respectively, which is consistent with previously published data [12,22]. Compounds **1e**, **1f**, and **2e** showed good activity and BA, **1b**, and **1h** showed moderate activity, whereas the other compounds, including UA, were considered inactive.

**Table 1:** **Anti-malarial activity of isolated and synthesized compounds against CQ-sensitive *Plasmodium falciparum *3D7**

**Series BA**		**Series UA**	
**Compound**	**IC**_**50**_^** a **^**(μM)**	**Compound**	**IC**_**50 **_**(μM)**
BA	18 ± 0.17	UA	36 ± 0.25
**1a**	> 100	**2a**	> 100
**1b**	29 ± 0.26	**2b**	71 ± 0.30
**1c**	44 ± 0.14	**2c**	72 ± 0.16
**1d**	70 ± 0.19	**2d**	ND
**1e**	5 ± 0.14	**2e**	7 ± 0.15
**1f**	8 ± 0.16	**2f**	> 100
**1g**	66 ± 0.25	**2g**	58 ± 0.13
**1h**	22 ± 0.17	**2h**	58 ± 0.32
**1i**	> 100	**2i**	> 100
**1j**	ND	**2j**	> 100

^a^: results expressed as mean ± standard error.

*ND* Not Determined;

*BA* Betulinic Acid.

*UA* Ursolic Acid.

### Cytotoxicity of active compounds on HEK293T mammalian cells

The IC_50_ of compounds **1e**, **1f** and **2e** for HEK293T was > 100 μM; therefore, none was cytotoxic in the tested conditions.

### Determination of changes in mitochondrial membrane potential (*ΔΨm*)

**Figure 3:**
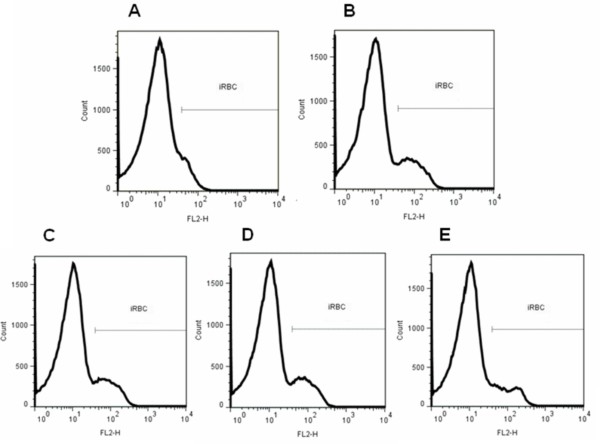
**Effect of derivatives on *ΔΨm.*** Representative histograms showing the effect of higher concentration (25 μM) of derivatives **1e** (3**C**), **1f** (3**D**) and **2e** (3**E**) on *ΔΨm* by FACS analysis. Negative control (3**B**) for the fluorescence intensity of parasites in the presence of 2 nM DiOC_6_(3) without any added inhibitors and with protonophore CCCP (5 μM) (3**A**).

Figure 3A presents the representative fluorescence histogram for iRBC displacement in incubation with CCCP at a concentration of 5 μM, indicating a differential probe location and ideal experimental conditions. Histograms obtained in control conditions (Figure 3B) and in the presence of the active compounds **1e**, **1f** and **2e** (Figure 3C, 3D and 3E, respectively) showed a normal mitochondrial membrane potential for the concentrations tested, in the 0.25 nM–25 μM range. These results suggest that the mechanism of anti-malarial action of these compounds in *P. falciparum* iRBC is distinct from that of atovaquone. Results were calculated from fluorescence intensity by flow cytometer setting.

### Inhibition of β-haematin formation

Inhibition percentages for the most active derivatives are reported in Table 2. CQ was significantly more efficient at inhibiting β-haematin formation than all tested compounds. No statistically significant differences were found between **1e** and **2e** (derived from BA and UA respectively). This suggests that the tested derivatives exert their anti-malarial effects via a mechanism of action distinct from that of CQ in the haemin detoxification pathway.

**Table 2:** Inhibition of β-haematin formation

**Compound**	**Concentration**	**Inhibition**^b^	**IC**_**50**_^ c^	**CQ**
	**(mM)**	**(%)**	**(mM)**	**index**^**a**^
Chloroquine	0.5 – 20	83 ± 1.07	3 ± 0.16	-
**1e**	0.5 – 19	25 ± 2.62	> 19	> 6.33
**1f**	0.5 – 20	14 ± 0.43	> 20	> 6.66
**2e**	0.5 – 19	26 ± 3.57	> 19	> 6.33

^a^ calculated as compound IC_50_/CQ IC_50_.

^b^ results are expressed as mean ± standard deviation at the highest tested concentration.

^c^ results are expressed as mean ± standard error.

## Discussion

In this study, semisynthetic compounds were obtained, derived from natural sources, with potential utility as anti-malarial agents, using a simple and inexpensive strategy.

The triterpenes BA and UA exhibited moderate anti-malarial activity *in vitro*[12,22]. Both compounds were obtained from natural sources with good yields. This work reported for the first time the semisynthesis of 18 BA and UA derivatives with ester substituents at C-3, designed to improve anti-malarial activity, reduce cytotoxicity and search for new targets. These derivatives were obtained using a single-step, inexpensive synthesis adequate for industrial-scale processing. The anti-malarial activities of the synthesized compounds were in the range of 5 and >100 μM against CQ-sensitive *P. falciparum* 3D7, with three compounds exhibiting good activity (**1e**, **1f** and **2e**). These three derivatives possessed a four-carbon side-chain and had anti-malarial activity three to five times greater than that of BA and UA. Some reports have reported a change in activity after modification on the triterpene carbon 3. The 3-O-acetyl ester of ursolic acid exhibited enhanced activity against CQ-resistant *P. falciparum* FcB1 (IC_50_ = 24.93 μM) as compared with UA (IC_50_ = 52.93 μM) [13]. Interestingly, the 3-O-acetyl ester of oleanolic acid, an isomer of UA, was also active against a multidrug-resistant *P. falciparum* K1 strain [23]. In another study, the 3-O-acetyl ester of lupeol containing a functional ester group at C-3 was more active than its precursor against CQ-resistant *P. falciparum* FCR-3 [24]. In view of these findings, modifications at the C-3 position of BA and UA were designed to evaluate the influence of side-chain length and presence of polar or lipophilic groups (such as carboxylic acids, aromatic rings or halogens) on their ability to impair parasite growth. In these series, acylated derivatives with shorter side-chains (**1e**, **1f** and **2e**) had improved anti-malarial activity (IC_50_ = 5, 8 and 7 μM, respectively). It was also obtained information on the presence of a second carboxylic acid group, as in derivatives **1d** (IC_50_ = 70 μM), **1g** (IC_50_ = 66 μM) and **2g** (IC_50_ = 58 μM); and the presence of a halogen, such as chlorine and fluorine, as in derivatives **1h** (IC_50_ = 22 μM), **1i** (IC_50_ >100 μM), **2h** (IC_50_ = 58 μM) and **2i** (IC_50_ >100 μM). These functional groups did not potentiate anti-malarial action.

Comparison of derivatives with the same substituents derived from series BA and series UA demonstrated that BA derivatives are generally more active against *P. falciparum* 3D7 strain than UA derivatives. The most active derivatives synthesized, **1e**, **1f** and **2e**, did not show any cytotoxicity against HEK293T cells at the tested concentration of 100 μM. This excellent result justifies potential *in vivo* experiments with these compounds in future. Follow-up studies with non-cytotoxic compounds are important, as adverse effects such as hypoglycaemia, cardiotoxicity, and gastrointestinal discomfort have been described with the anti-malarials: QN, halofantrine, and CQ/proguanil [22].

The molecular mechanism of action of triterpenes such as BA and UA is still poorly understood. Previous research has suggested that the mechanism of action of UA and its derivatives could be similar to that of CQ, i.e. inhibition of β-haematin formation [13,25]. It was chose to assess this mechanism of action and another mechanism that has been suggested for an important anti-malarial agent, atovaquone: an inhibitory effect on *ΔΨm*. The *ΔΨm* consists of chemical and electrical components generated by electron transport chain enzymes, and its determination is widely used to characterize cellular metabolism, viability and apoptosis [26]. The *ΔΨm* assay was performed for the more active derivatives, **1e**, **1f** and **2e**, by accumulation of the lipophilic cationic fluorescent probe DiOC_6_(3). When DiOC_6_(3) is incubated with iRBC, it diffuses into cells and concentrates several orders of magnitude into negative-inside mitochondria. Probe accumulation into parasite mitochondria is dependent on the presence of a membrane potential, collapse of which will result in diffusion of the probe out of the mitochondria, resulting in signal dissipation. The tested derivatives did not exhibit histogram displacement, as observed after incubation with CCCP, indicating dissipation of the membrane potential and abolition of probe accumulation. This experiment showed that, unlike CCCP, the tested compounds were not able to collapse *ΔΨm* (Figure 3).

Derivatives **1e**, **1f** and **2e** were also evaluated for inhibition of β-haematin formation, and displayed less inhibitory activity than that of CQ. This assay is based on the ability of *Plasmodium* to use haemoglobin as a source of amino acids, resulting in the formation of potentially toxic ferriprotoporphyrin IX. CQ and other anti-malarial drugs act by inhibiting ferriprotoporphyrin IX detoxification through haem polymerization. The action of these anti-malarial drugs on β-haematin formation takes place during the intra-erythrocytic phase of the parasite, within the food vacuole. The parasite converts haem into the malarial pigment haemozoin [15]. Derivatives **1e** and **2e** derivatives exhibited similar inhibition percentages (25% and 26% respectively), whereas **1f** displayed less inhibitory activity (14%) than the other tested derivatives. Thus, it was observed that modification of carbon 3 plays an important role in the inhibition of β-haematin formation.

## Conclusion

In this study, 18 new derivatives of betulinic acid (series BA) and ursolic acid (series UA) were synthesized with the purpose of investigating the importance of substituents at carbon 3 for anti-malarial activity against *P. falciparum* 3D7. Derivatives **1e**, **1f** and **2e** exhibited good anti-malarial activity in the micromolar range. Derivatives of series BA were generally more active against *P. falciparum* 3D7 than compounds derived from series UA, with a three- to five-fold difference in activity in relation to their respective aglycones. Furthermore, **1e**, **1f** and **2e** did not exhibit any cytotoxicity against a HEK293T cell line at 100 μM. Regarding mechanism of action, these triterpene derivatives was weaker inhibit β-haematin formation than CQ and did not act on *ΔΨm*. Further assays are required to elucidate the pathways whereby these anti-malarial compounds exert their effects. This study can contribute to the design and development of potent anti-malarial compounds derived from natural sources.

## Abbreviations

BA: Betulinic acid; CQ: Chloroquine; OD: Optical density; UA: Ursolic acid

## Competing interests

The authors declare that they have no competing interests.

## Authors’ contributions

SCBG, GG, CRSG and GNSS designed and coordinated the research. GNSS and NRGM obtained the betulinic and ursolic acids, and corresponding derivatives. GNSS and MN performed the experiments for the anti-malarial activity. GNSS and MSM performed the cytotoxicity assays. GNSS, DCS and LNC were responsible for the assay on Δ*Ψm* and inhibition of β-haematin formation. GNSS and LNC drafted the manuscript. SCBG, GG and CRSG corrected the manuscript. All authors approved the final manuscript.

## Supplementary Material

Additional file 1 **Extraction of BA and UA.** Description: The data provided represent the extraction of betulinic and ursolic acids. Click here for file

Additional file 2 **Identification of synthesized derivatives.** Description: The data provided represent the identification of the synthesized derivatives. Click here for file
