# Supplementary material for: Two series of new semisynthetic triterpene derivatives: differences in anti-malarial activity, cytotoxicity and mechanism of action
Source: Malar J. 2013 Mar 9;12:89. doi: 10.1186/1475-2875-12-89 (PMC3616855; doi:10.1186/1475-2875-12-89)
Supplement: Additional file 1 — Extraction of BA and UA. Description: The data provided represent the extraction of betulinic and ursolic acids. [file 1475-2875-12-89-S1.doc]

**Additional file**

**Additional file 1**

**File format: Doc**

**Title: Extraction of BA and UA**

**Description: The data provided represent the extraction of betulinic and ursolic acids**

Dried-powdered barks of *P. acerifolia* (140 g) were exhaustively extracted with ethanol (400 mL) by reflux. Briefly, the EtOH extract was dissolved in H_2_O (100 mL) and extracted successively with dichloromethane (100 mL 3 x) for removal of waxy material and ethyl acetate (100 mL 3 x). After evaporation under vacuum (40°C) following crystallization using methanol, it was obtained BA. The peels of *M. domestica* were dried and after submitted to extraction using H_2_O (1 L 3 x) by decoction to remove glycons portion following extraction with EtOH (400 mL) by reflux. The extract was evaporated under vacuum (40°C), yielding a residue that was chromatographed over silica gel using CH_2_Cl_2_ as eluent to obtain UA.
