# Supplementary material for: Two series of new semisynthetic triterpene derivatives: differences in anti-malarial activity, cytotoxicity and mechanism of action
Source: Malar J. 2013 Mar 9;12:89. doi: 10.1186/1475-2875-12-89 (PMC3616855; doi:10.1186/1475-2875-12-89)
Supplement: Additional file 2 — Identification of synthesized derivatives. Description: The data provided represent the identification of the synthesized derivatives. [file 1475-2875-12-89-S2.doc]

**Additional file 2**

**File format: Doc**

**Title: Identification of synthesized derivatives**

**Description: The data provided represent the identification of the synthesized derivatives**

**3-*O*-hexanoylbetulinic acid (1a)**

The compound **1a** was prepared using caproic anhydride. White Powder, yield = 47%. IR (ATR, cm^-1^): 2929 (OH acid); 2867(C-H); 1725 (C=O ester); 1692 (C=O acid); 1453 (C-O). ^1^H NMR (300 MHz, CDCl_3_), δ (ppm): 0.89 (t, 3H, CH_3_-36); 0.93 (s, 3H, CH_3_-26 and CH_3_-27); 0.97 (s, 3H, CH_3_-23 and CH_3_-24); 1.03 (s, 3H, CH_3_-25);1.33 (m, 2H, CH_2_-34); 1.36 (m, 2H, CH_2_-35); 1.40 (d, 2H; CH_2_-7); 1.44 (t, 2H, CH_2_-16); 1.50 (m, 2H, CH_2_-33);1.55 (d, 1H, CH-5; CH-13); 1.61 (t, 1H, CH-9); 1.64 (br s, 3H, CH_3_-30); 1.65 (m, 2H, CH_2_-11); 1.69 (t, 2H, CH_2_-1, CH_2_-6, CH_2_-12); 1.95 (m, 2H, CH_2_-21; CH_2_-22); 1.99 (t, 2H, CH_2_-15); 2.28 (m, 2H, CH_2_-32); 2.31 (ddd, 1H, CH-19); 4.47 (s, 1H, CH-29b); 4.50 (dd, 1H, CH-3); 4.61 (s, 1H, CH-29a). ^13^C NMR (75 MHz, CDCl_3_), δ (ppm): 14 (C-27); 15 (C-36); 15.3 (C-26); 16.6 (C-25); 18 (C-6); 19 (C-30); 20.8 (C-11); 22.2 (C-23; C-24); 22.2 (C-35); 23.7 (C-2); 24.8 (C-33); 25.4 (C-12); 29.7 (C-15); 30.6 (C-21); 31.3 (C-34); 32 (C-16); 33 (C-32); 34.2 (C-7); 37 (C-10); 37.1 (C-22); 37.8 (C-4); 38.3 (C-13); 38.4 (C-1); 40.5 (C-8); 42.4 (C-14); 46.9 (C-19); 49.3 (C-18); 50.4 (C-9); 55.4 (C-5); 56.4 (C-17); 80.6 (C-3); 150.3 (C-20); 109.7 (C-29); 173.7 (C-31); 182.1 (C-28). HRMS (ESI-MS, m/z); [M+Na]^+^ calcd. for C_36_H_58_O_4_Na: 577.8564; found: 577.4219. Mp: 237-238ºC

**3-*O*-pentanoylbetulinic acid (1b)**

The compound **1b** was prepared using pentanoic anhydride. Yellow powder, yield = 50%. IR (ATR, cm^-1^): 2937 (OH acid); 2866 (C-H); 1728 (C=O ester); 1693 (C=O acid); 1463 (C-O). ^1^H NMR (300 MHz, CDCl_3_), δ (ppm): 0.89 (t, 3H, CH_3_-35); 0.93 (s, 3H, CH_3_-26 and CH_3_-27); 0.98 (s, 3H, CH_3_-23 and CH_3_-24); 1.05 (s, 3H, CH_3_-25); 1.34 (m, 2H, CH_2_-34); 1.45 (t, 2H, CH_2_-16); 1.54 (d, 1H, CH-5); 1.55 (m, 1H CH-13); 1.56 (m, 2H, CH_2_-33); 1.58 (d, 2H; CH_2_-7); 1.62 (t, 1H, CH-9); 1.66 (br s, 3H, CH_3_-30); 1.67 (t, 2H, CH_2_-1); 1.68 (m, 2H, CH_2_-11); 1.69 (m, 2H, CH_2_-12);1.71 (m, 2H, CH_2_-6); 1.84 (t, 1H, CH-18); 1.91 (m, 2H, CH_2_-2); 1.94 (t, 2H, CH_2_-22);1.95 (m, 2H, CH_2_-21); 1.99 (t, 2H, CH_2_-15); 2.22 (t, 2H, CH_2_-32); 2.91 (ddd, 1H, CH-19); 4.44 (dd, 1H, CH-3); 4.54 (s, 1H, CH-29b); 4.66 (s, 1H, CH-29a). ^13^C NMR (75 MHz, CDCl_3_), δ (ppm): 14 (C-35); 15.3 (C-27); 16 (C-26); 16.5 (C-25); 18.1 (C-6); 19.3 (C-30); 20.9 (C-11); 22.3 (C-23; C-24); 23.7 (C-2); 25.4 (C-12); 27.2 (C-34); 27.9 (C-33); 29.7 (C-15); 30.6 (C-21); 32.1 (C-16); 34.2 (C-7); 34.5 (C-32); 37 (C-10); 37.1 (C-22); 37.8 (C-4); 38.4 (C-1; C-13); 40.7 (C-8); 42.4 (C-14); 46.9 (C-19); 49.3 (C-18); 50.3 (C-9); 55.4 (C-5); 56.4 (C-17); 80.6 (C-3); 109.7 (C-29); 150.3 (C-20); 173.7 (C-31); 181.8 (C-28). HRMS (ESI-MS, m/z); [M+Na]^+^ calcd. for C_35_H_56_O_4_Na: 563.8264; found: 563.4086. Mp: 259-262°C

**3-*O*-isovalerylbetulinic acid (1c)**

The compound **1c** was prepared using isovaleric anhydride. White powder, yield = 45%. IR (ATR, cm^-1^): 2938 (OH acid); 2869 (C-H); 1728 (C=O ester); 1695 (C=O acid); 1464 (C-O). ^1^H NMR (300 MHz, CDCl_3_), δ (ppm): (t, 2H, CH_2_-1), 1.90 (m, 2H, CH_2_-2); 4.40 (dd, 1H, CH-3); 1.53 (d, 1H, CH-5); (m, 2H, CH_2_-6); 1.58 (d, 2H; CH_2_-7); 1.65 (t, 1H, CH-9); 1.60 (m, 2H, CH_2_-11); 1.62 (m, 2H, CH_2_-12);1.54 (m, 1H CH-13); 1.93 (t, 2H, CH_2_-15); 1.44 (t, 2H, CH_2_-16); 1.91 (t, 1H, CH-18); 2.90 (ddd, 1H, CH-19); 1.89 (m, 2H, CH_2_-21); 1.61 (t, 2H, CH_2_-22); 0.97 (s, 3H, CH_3_-23 and CH_3_-24); 1.09 (s, 3H, CH_3_-25); 0.92 (s, 3H, CH_3_-26 and CH_3_-27); 4.67 (s, 1H, CH-29a); 4.54 (s, 1H, CH-29b); 1.65 (br s, 3H, CH_3_-30); 2.22 (d, 2H, CH_2_-32); 2.18 (m, H, CH-33); 1.10 (d, 3H, CH_3_-34 and CH_3_-35). ^13^C NMR (75 MHz, CDCl_3_), δ (ppm): 14.6 (C-27); 16.0 (C-26); 16.5 (C-25); 18.1 (C-6); 19.3 (C-30); 20.8 (C-11); 22.3 (C-23 and C-24); 22.4 (C-34 and C-35); 23.7 (C-2); 25.4 (C-12); 27.9 (C-33); 29.7 (C-15); 30.6 (C-21); 32.1 (C-16); 34.2 (C-7); 37.0 (C-10 and C-22); 37.1 (C-4); 37.7 (C-13); 38.4 (C-1); 40.7 (C-8); 42.4 (C-14); 44.0 (C-32); 46.9 (C-19); 49.3 (C-18); 50.4 (C-9); 55.4 (C-5); 56.4 (C-17); 80.6 (C-3); 109.6 (C-29); 150.3 (C-20); 172.9 (C-31); 181.9 (C-28). HRMS (ESI-MS, m/z); [M+Na]^+^ calcd. for C_35_H_56_O_4_Na: 563.8264; found: 563.4088. Mp: 268-270°C

**3-*O*-methylmalenylbetulinic acid (1d)**

The compound **1d** was prepared using methylmaleic anhydride. Yellow powder, yield = 24%. IR (ATR, cm^-1^): 2942 (OH acid); 1764 (C=O ester); 1711 (C=O acid); 1687 (C=O acid); 1446 (C-O). ^1^H NMR (300 MHz, CDCl_3_), δ (ppm): 0.89 (s, 3H, CH_3_-23, CH_3_-24, CH_3_-26 and CH_3_-27); 1.05 (s, 3H, CH_3_-25); 1.41 (d, 2H; CH_2_-7); 1.45 (t, 2H, CH_2_-16); 1.49 (d, 1H, CH-5); 1.55 (br s, 3H, CH_3_-30); 1.86 (m, 2H, CH_2_-2, CH_2_-6); 1.86 (t, 1H, CH-18); 2.08 (t, 2H, CH_2_-15, CH_2_-21 and CH_2_-22); 2.13 (s, 3H, CH_3_-35); 2.58 (ddd, 1H, CH-19); 4.29 (dd, 1H, CH-3); 4.46 (s, 1H, CH-29b); 4.49 (s, 1H, CH-29a); 5.16 (s, 2H, CH_2_-33). ^13^C NMR (75 MHz, CDCl_3_), δ (ppm): 15.8 (C-35 and C-26); 17.0 (C-25 and C-27); 18.3 (C-6 and C-30); 20.3 (C-11); 21.3 (C-23 and C-24); 23.6 (C-2); 23.8 (C-12); 29.6 (C-15 and C-21); 29.9 (C-16); 37.1 (C-1, C-4, C-7 and C-10); 38.3 (C-13); 38.6 (C-22); 39.7 (C-8); 42.0 (C-14); 47.5 (C-19); 49.1 (C-18); 52.6 (C-9); 55.4 (C-5 and C-17); 81.7 (C-3); 138.1 (C-33); 171.8 (C-34); 178.1 (C-31); 184.2 (C-28). HRMS (ESI-MS, m/z); [M+Na]^+^ calcd. for C_35_H_52_O_6_Na: 591.7964; found: 591.3677. Mp: 268°C

**3-*O*-butanoylbetulinic acid (1e)**

The compound **1e** was prepared using butyric anhydride. Yellow powder, yield = 25%. IR (ATR, cm^-1^): 2936 (OH acid); 2872 (C-H); 1730 (C=O ester); 1696 (C=O acid); 1463 (C-O). ^1^H NMR (300 MHz, CDCl_3_), δ (ppm): 0.90 (s, 3H, CH_3_-26 and CH_3_-27); 0.96 (t, 3H, CH_3_-34); 0.98 (s, 3H, CH_3_-23 and CH_3_-24); 1.04 (s, 3H, CH_3_-25); 1.45 (t, 2H, CH_2_-16); 1.50 (d, 1H, CH-5); 1.54 (m, 1H CH-13); 1.57 (m, 2H, CH_2_-33); 1.59 (d, 2H; CH_2_-7); 1.62 (br s, 3H, CH_3_-30); 1.86 (t, 1H, CH-18); 1.88 (t, 2H, CH_2_-1), 1.90 (m, 2H, CH_2_-2); 1.91 (m, 2H, CH_2_-21); 1.95 (t, 2H, CH_2_-22); 1.96 (t, 2H, CH_2_-15); 2.23 (t, 2H, CH_2_-32); 2.91 (ddd, 1H, CH-19); 4.43 (dd, 1H, CH-3); 4.54 (s, 1H, CH-29b); 4.66 (s, 1H, CH-29a). ^13^C NMR (75 MHz, CDCl_3_), δ (ppm): 13 (C-34); 15 (C-27); 16 (C-26); 16.5 (C-25); 18.1 (C-6); 18.6 (C-33); 19.3 (C-30); 20.8 (C-11); 23.6 (C-2); 25.4 (C-12); 27.9 (C-23; C-24); 29.7 (C-15); 30.6 (C-21); 32.1 (C-16); 34.2 (C-7); 36.7 (C-22; C-32); 37.0 (C-10); 37.1 (C-4); 37.8 (C-13); 38.4 (C-1); 40.7 (C-8); 42.4 (C-14); 46.9 (C-19); 49.3 (C-18); 50.4 (C-9); 55.4 (C-17); 56.4 (C-5); 80.6 (C-3); 150.3 (C-20); 109.7 (C-29); 173.5 (C-31); 181.5 (C-28). HRMS (ESI-MS, m/z); [M+Na]^+^ calcd. for C_34_H_54_O_4_Na: 549.8064; found: 549.3933. Mp: 252-254°C

**3-*O*-isobutyrylbetulinic acid (1f)**

The compound **1f** was prepared using isobutyric anhydride. Yellow powder, yield = 62%. IR (ATR, cm^-1^): 2934 (OH acid); 2867 (C-H); 1729 (C=O ester); 1701 (C=O acid); 1465 (C-O). ^1^H NMR (300 MHz, CDCl_3_), δ (ppm): 0.90 (s, 3H, CH_3_-26 and CH_3_-27); 0.98 (s, 3H, CH_3_-23 and CH_3_-24); 1.04 (s, 3H, CH_3_-25); 1.07 (d, 3H, CH_3_-33 and CH_3_-34); 1.40 (d, 2H; CH_2_-7); 1.45 (t, 2H, CH_2_-16); 1.50 (br s, 3H, CH_3_-30); 1.51 (m, 2H, CH_2_-12); 1.53 (d, 1H, CH-5); 1.54 (m, 1H CH-13); 1.55 (t, 1H, CH-9); 1.58 (m, 2H, CH_2_-11); 1.62 (t, 2H, CH_2_-1); 1.84 (m, 2H, CH_2_-6); 1.86 (t, 1H, CH-18); 1.90 (m, 2H, CH_2_-2); 1.96 (m, 2H, CH_2_-21); 1.95 (t, 2H, CH_2_-22); 1.98 (t, 2H, CH_2_-15); 2.44 (m, 1H, CH_2_-32); 2.51 (ddd, 1H, CH-19); 4.53 (dd, 1H, CH-3); 4.54 (s, 1H, CH-29b); 4.66 (s, 1H, CH-29a). ^13^C NMR (75 MHz, CDCl_3_), δ (ppm): 14.6 (C-27); 15.9 (C-26); 16.0 (C-25); 18.1 (C-6); 18.8 (C-33 and C-34); 19.2 (C-30); 20.8 (C-11); 23.6 (C-2, C-23 and C-24); 25.4 (C-12); 29.6 (C-15); 30.5 (C-21); 32.1 (C-16); 34.2 (C-32); 34.4 (C-7); 36.9 (C-1); 37.1 (C-10); 37.9 (C-4); 38.3 (C-13 and C-22); 40.6 (C-8); 42.4 (C-14); 46.8 (C-19); 49.2 (C-18); 50.3 (C-9); 56.3 (C-5 and C-17); 77.2 (C-3); 109.6 (C-29); 150.2 (C-20); 176.7 (C-31); 181.4 (C-28). HRMS (ESI-MS, m/z); [M+Na]^+^ calcd. for C_34_H_54_O_4_Na: 549.8064; found: 549.3925. Mp: 260-262°C

**3-*O*-succinylbetulinic acid (1g)**

The compound **1g** was prepared using succinyl anhydride. Yellow powder, yield = 91%. IR (ATR, cm^-1^): 2927 (OH acid); 2869 (C-H); 1685 (C=O acid); 1730 (C=O ester). ^1^H NMR (300 MHz, CDCl_3_), δ (ppm): 0.90 (s, 3H, CH_3_-23 and CH_3_-24); 0.94 (s, 3H, CH_3_-26 and CH_3_-27); 1.14 (s, 3H, CH_3_-25); 1.45 (t, 2H, CH_2_-16); 1.50 (br s, 3H, CH_3_-30); 1.54 (d, 1H, CH-5); 1.58 (d, 2H; CH_2_-7); 1.62 (t, 1H, CH-9); 1.88 (t, 2H, CH_2_-1); 1.90 (t, 1H, CH-18); 1.92 (m, 2H, CH_2_-2, CH_2_-21, CH_2_-22); 2.55 (t, 2H, CH_2_-32); 2.59 (t, 2H, CH_2_-33); 2.61 (ddd, 1H, CH-19); 4.53 (dd, 1H, CH-3); 4.60 (s, 1H, CH-29b); 4.70 (s, 1H, CH-29a). ^13^C NMR (75 MHz, CDCl_3_), δ (ppm): 14 (C-27); 15.3 (C-26); 16.6 (C-25); 18 (C-6);19 (C-30); 20 (C-11); 22 (C-23; C-24); 23 (C-2); 25.4 (C-12); 26 (C-13); 28 (C-32; C-33); 29.6 (C-15); 31.3 (C-21); 32.6 (C-16); 34 (C-7); 36.6 (C-22); 37 (C-10); 37.5 (C-4); 38 (C-1); 39.6 (C-13); 41.3 (C-8); 42.6 (C-14); 46.6 (C-19); 49 (C-18); 51.3 (C-9) 55.3 (C-5); 56.6 (C-17); 81.3 (C-3); 110 (C-29); 150.6 (C-20); 172.6 (C-31); 174 (C-34); 181.3 (C-28). HRMS (ESI-MS, m/z); [M+Na]^+^ calcd. for C_34_H_52_O_6_Na: 579.7864; found: 579.3654. Mp: 245-248ºC

**3-*O*-dichloroacylbetulinic acid (1h)**

The compound **1h** was prepared using dichloroacetic anhydride. Yellow powder, yield = 61%. IR (ATR, cm^-1^): IR (ATR, cm^-1^): 2936 (OH acid); 2872 (C-H); 1742 (C=O ester); 1685 (C=O acid); 1451 (C-O); 817 (C-Cl). ^1^H NMR (300 MHz, CDCl_3_), δ (ppm): 0.90 (s, 3H, CH_3_-26 and CH_3_-27); 0.99 (s, 3H, CH_3_-23 and CH_3_-24); 1.14 (s, 3H, CH_3_-25); 1.44 (t, 2H, CH_2_-16); 1.47 (d, 2H; CH_2_-7); 1.51 (d, 1H, CH-5); 1.55 (m, 1H CH-13); 1.62 (m, 2H, CH_2_-12); 1.67 (br s, 3H, CH_3_-30); 1.89 (t, 1H, CH-18); 1.92 (m, 2H, CH_2_-21); 1.93 (t, 2H, CH_2_-22); 1.97 (t, 2H, CH_2_-15); 2.90 (ddd, 1H, CH-19); 4.52 (dd, 1H, CH-3); 4.54 (s, 1H, CH-29b); 4.67 (s, 1H, CH-29a); 5.86 (s, 2H, CH_2_-32). ^13^C NMR (75 MHz, CDCl_3_) δ (ppm): 14 (C-27); 16.1 (C-26) ; 16.3 (C-25); 18.0 (C-6); 19.30 (C-30); 20.9 (C-11); 23.2 (C-2; C-23; C-24); 25.4 (C-12); 29.6 (C-15); 30.5 (C-21); 32.1 (C-16); 34.2 (C-7); 37.0 (C-4); 37.1 (C-10);  38.2 (C-13); 38.4 (C-1); 40.7 (C-8); 42.4 (C-14); 46.9 (C-19); 49.2 (C-18); 50.4 (C-9); 55.4 (C-5); 56.4 (C-17); 64.8 (C-32); 84.9 (C-3); 109.7 (C-29); 150.3 (20); 164.3 (C-31); 182.2 (C-28). Anal. Calcd. for C_32_H_48_Cl_2_O_4_: Cl, 12.49; found: Cl, 12.23. Mp: 269-270ºC

**3-*O*-trifluoraceylbetulinic acid (1i)**

The compound **1i** was prepared using Bis(trifluoroacetic) anhydride. Yellow powder, yield = 100%. IR (ATR, cm^-1^): 2938 (OH acid); 2867(C-H); 1774 (C=O ester); 1694 (C=O acid); 1158 (C-F). ^1^H NMR (300 MHz, CDCl_3_), δ (ppm): 0.90 (s, 3H, CH_3_-26 and CH_3_-27); 0.99 (s, 3H, CH_3_-23 and CH_3_-24); 1.09 (s, 3H, CH_3_-25);1.46 (t, 2H, CH_2_-16); 1.50 (d, 1H, CH-5); 1.54 (m, 1H CH-13); 1.58 (d, 2H; CH_2_-7); 1.62 (t, 1H, CH-9); 1.65 (m, 2H, CH_2_-11); 1.66 (br s, 3H, CH_3_-30); 1.89 (t, 2H, CH_2_-1); 1.90 (t, 1H, CH-18); 1.92 (m, 2H, CH_2_-21, CH_2_-22); 2.18 (t, 2H, CH_2_-15); 2.18 (ddd, 1H, CH-19); 4.57 (s, 1H, CH-29b); 4.60 (s, 1H, CH-29a); 4.66 (dd, 1H, CH-3). ^13^C NMR (75 MHz, CDCl_3_), δ (ppm): 14.6 (C-27); 15.9 (C-26); 16.0 (C-25);18 (C-6); 19.5 (C-30); 20.4 (C-11); 22 (C-23; C-24); 23 (C-2); 25.3 (C-12); 29.6 (C-15); 30.5 (C-21); 32.1 (C-16); 34.1 (C-7); 37.0 (C-10); 37.1 (C-22); 38.0 (C-4); 38.2 (C-13); 38.3 (C-1); 40.7 (C-8); 42.4 (C-14); 46.9 (C-19); 49.2 (C-18); 50.4 (C-9); 55.3 (C-5); 56.3 (C-17); 86.6 (C-3); 106.7 (C-32); 109.7 (C-29); 150.3 (C-20); 157.4 (C-31); 181.4 (C-28). HRMS (ESI-MS, m/z); [M-H+OH]^+^ calcd. for C_32_H_46_F_3_O_4_: 569.734; found: 569.3663. Mp: 234-237ºC

**3-*O*-hexanoylursolic acid (2a)**

The compound **2a** was prepared using caproic anhydride. White powder, yield = 50%. IR (ATR, cm^-1^): 2924 (OH acid); 2859 (C-H); 1690 (C=O acid); 1728 (C=O ester); 1460 (C-O). ^1^H NMR  (300 MHz, CDCl_3_ ), δ (ppm): 0.68 (t, H, CH-5); 0.74 (s, 3H, CH_3_-25); 0.78 (s, 3H, CH_3_-24); 0.81 (d, 3H, CH_3_-30); 0.850 (s, 3H, CH_3_-26); 0.855 (t, 3H, CH_3_-36); 0.87 (s, 3H, CH_3_-23); 0.88 (t, 2H, CH_2_-11); 1.01 (m, H, CH-20); 1.11 (s, 3H, CH_3_-27); 1.13 (d, 3H, CH_3_-29); 1.22 (m, 2H, CH_2_-7); 1.26 (m, 2H, CH_2_-35); 1.27 (m, 2H, CH_2_-34); 1.30 (m, 2H, CH_2_-2); 1.38 (m, H, CH-19); 1.45 (s, H, CH-9); 1.47 (t, 2H, CH_2_-21); 1.48 (m, 2H, CH_2_-6); 1.54 (m, 2H, CH_2_-33); 1.57 (t, 2H, CH_2_-15); 1.58 (t, 2H, CH_2_-1); 1.83 (t, 2H, CH_2_-16); 1.86 (dd, 2H, CH_2_-22); 2.13 (d, H, CH-18); 2.25 (t, 2H, CH_2_-32); 4.43 (dd, 1H, CH-3); 5.23 (tl, H, CH-12). ^13^C NMR (75 MHz, CDCl_3_), δ (ppm): 14.1 (C-36); 15.7 (C-25); 16.9 (C-26 and C-29); 18.3 (C-6); 21.3 (C-30); 22.5 (C-23, C-24 and C-35); 23.5 (C-2, C-11 and C-27); 23.8 (C-16); 24.5 (C-33); 28.1 (C-15); 30.8 (C-21); 31.5 (C-7 and C-34); 33.0 (C-32); 36.9 (C-10 and C-22); 37.1 (C-4); 37.9 (C-20); 38.4 (C-1 and C-19); 39.2 (C-8); 42.1 (C-14); 47.6 (C-9 and C-17); 52.7 (C-18); 55.5 (C-5); 80.8 (C-3); 125.9 (C-12); 138.1 (C-13); 173.9 (C-31); 184.5 (C-28). HRMS (ESI-MS, m/z); [M+Na]^+^ calcd. for C_36_H_58_O_4_Na: 577.8579; found: 577.4236. Mp: 242-244ºC

**3-*O*-pentanoylursolic acid (2b)**

The compound **2b** was prepared using pentanoic anhydride. Yellow powder, yield = 56%. IR (ATR, cm^-1^): 2925 (OH acid); 2871 (C-H); 1729 (C=O ester); 1688 (C=O acid); 1460 (C-O). ^1^H NMR (300 MHz, CDCl_3_ ), δ (ppm): 0.67 (t, H, CH-5); 0.73 (s, 3H, CH_3_-25); 0.75 (s, 3H, CH_3_-24); 0.81 (d, 3H, CH_3_-30); 0.87 (s, 3H, CH_3_-26); 0.90 (d, 3H, CH_3_-29); 0.92 (s, 3H, CH_3_-23); 0.93 (t, 2H, CH_2_-11); 0.95 (t, 3H, CH_3_-35); 1.06 (s, 3H, CH_3_-27); 1.22 (m, 2H, CH_2_-7); 1.31 (m, 2H, CH_2_-34); 1.33 (m, 2H, CH_2_-2); 1.34 (m, H, CH-19); 1.45 (t, 2H, CH_2_-21); 1.47 (m, 2H, CH_2_-6); 1.48 (s, H, CH-9); 1.56 (t, 2H, CH_2_-15); 1.59 (t, 2H, CH_2_-1); 1.60 (m, 2H, CH_2_-33);1.82 (t, 2H, CH_2_-16); 1.86 (dd, 2H, CH_2_-22); 2.14 (d, H, CH-18); 2.30 (t, 2H, CH_2_-32); 4.40 (dd, 1H, CH-3); 5.17 (tl, H, CH-12). ^13^C NMR (75 MHz, CDCl_3_), δ (ppm): 14 (C-27 and C-35); 15.3 (C-25); 16.8 (C-29); 18 (C-26); 18.1 (C-6); 21.1 (C-30); 22.3 (C-23; C-24; C-34); 23.4 (C-11); 23.6 (C-2); 24.0 (C-16); 27.2 (C-15); 28 (C-33); 30.4 (C-21); 32.7 (C-7); 34.5 (C-32); 36.7 (C-22); 36.9 (C-10); 37.7 (C-4); 38.2 (C-1); 38.8 (C-19); 39.0 (C-20); 39.5 (C-8); 41.9 (C-14); 47.4 (C-9); 48.0 (C-17); 52.5 (C-18); 55.3 (C-5); 80.6 (C-3); 125.7 (C-12); 137.9 (C-13); 173.7 (C-31); 183.7 (C-28). HRMS (ESI-MS, m/z); [M+Na]^+^ calcd. for C_35_H_56_O_4_Na: 563.8279; found: 563.4065. Mp: 183-186ºC

**3-*O*-isovalerylursolic acid (2c)**

The compound **2c** was prepared using isovaleric anhydride. White powder, yield = 40%. IR (ATR, cm^-1^): 2925 (OH acid); 2870 (C-H); 1728 (C=O ester); 1688 (C=O acid); 1462 (C-O). ^1^H NMR (300 MHz, CDCl_3_ ), δ (ppm): 0.65 (t, H, CH-5); 0.70 (s, 3H, CH_3_-25); 0.78 (s, 3H, CH_3_-24); 0.82 (d, 3H, CH_3_-30); 0.87 (s, 3H, CH_3_-26); 0.89 (d, 3H, CH_3_-29); 0.89 (m, H, CH-20); 0.95 (t, 2H, CH_2_-11); 0.95 (s, 3H, CH_3_-23); 1.00 (d, 6H, CH_3_-34 and CH_3_-35); 1.05 (s, 3H, CH_3_-27); 1.18 (m, 2H, CH_2_-7); 1.23 (m, 2H, CH_2_-2); 1.41 (m, H, CH-19); 1.45 (s, H, CH-9); 1.45 (m, 2H, CH_2_-6); 1.50 (t, 2H, CH_2_-21); 1.55 (t, 4H, CH_2_-1 and CH_2_-15); 1.85 (t, 4H, CH_2_-16 and CH_2_-22); 2.09 (d, 3H, CH-18 and CH_2_-32); 2.09 (m, H, CH-33); 4.46 (dd, 1H, CH-3); 5.22 (tl, H, CH-12). ^13^C NMR (75 MHz, CDCl_3_), δ (ppm): 15.4 (C-25); 16.7 (C-26); 16.9 (C-29); 18.1 (C-6); 21.1 (C-30); 22.3 (C-23 and C-24); 22.4 (C-34 and C-35); 23.2 (C-11); 23.4 (C-27); 23.5 (C-2); 24.0 (C-16); 25.9 (C-33); 27.9 (C-15); 30.6 (C-21); 32.8 (C-7); 36.7 (C-22); 36.9 (C-10); 37.6 (C-4); 38.2 (C-1); 38.8 (C-20); 39.0 (C-19); 39.5 (C-8); 41.8 (C-14); 43.9 (C-32); 47.4 (C-9); 47.9 (C-17); 52.4 (C-18); 55.3 (C-5); 80.6 (C-3); 125.7 (C-12); 137.9 (C-13); 172.9 (C-31); 184.0 (C-28). HRMS (ESI-MS, m/z); [M+Na]^+^ calcd. for C_35_H_56_O_4_Na: 563.8279; found: 563.4061. Mp: 133-136ºC

**3-*O*-butanoylursolic acid (2e)**

The compound **2e** was prepared using butyric anhydride. Yellow powder, yield = 30%. IR (ATR, cm^-1^): 2928 (OH acid); 2873 (C-H); 1729 (C=O ester); 1689 (C=O acid); 1457 (C-O). ^1^H NMR (300 MHz, CDCl_3_ ), δ (ppm): 0.68 (t, H, CH-5); 0.70 (s, 3H, CH_3_-25); 0.75 (s, 3H, CH_3_-24); 0.80 (d, 3H, CH_3_-30); 0.88 (s, 3H, CH_3_-26); 0.90 (m, H, CH-20); 0.92 (t, 2H, CH_2_-11); 0.94 (s, 3H, CH_3_-23); 1.00 (t, 3H, CH_3_-34); 1.05 (s, 3H, CH_3_-27); 1.23 (m, 2H, CH_2_-7); 1.26 (m, 2H, CH_2_-2); 1.43 (m, H, CH-19); 1.48 (m, 2H, CH_2_-6); 1.49 (s, H, CH-9); 1.50 (t, 2H, CH_2_-21); 1.57 (t, 2H, CH_2_-15); 1.58 (t, 2H, CH_2_-1); 1.63 (m, 2H, CH_2_-33); 1.83 (t, 2H, CH_2_-16); 1.85 (dd, 2H, CH_2_-22); 2.18 (d, H, CH-18); 2.23 (t, 2H, CH_2_-32); 4.45 (dd, 1H, CH-3); 5.28 (tl, H, CH-12). ^13^C NMR (75 MHz, CDCl_3_), δ (ppm): 14 (C-25); 15.3 (C-36); 16.6 (C-26); 16.9 (C-29); 18.1 (C-6); 18.6 (C-30); 21.1 (C-23; C-24); 23.2 (C-11); 23.5 (C-2;C-35); 24.0 (C-16;C-33); 28.0 (C-15); 30.5 (C-21); 32.8 (C-7; C-32; C-34); 36.7 (C-22); 36.9 (C-10); 37.7 (C-4); 38.2 (C-1); 38.8 (C-19); 39.0 (C-20); 39.5 (C-8); 41.9 (C-14); 47.3 (C-9); 47.9 (C-17); 52.5 (C-18); 55.3 (C-5); 80.5 (C-3); 125.7 (C-12); 137.9 (C-13); 173.5 (C-31); 184 (C-28). HRMS (ESI-MS, m/z); [M-H]^-^ calcd. for C_34_H_54_O_4_: 525.8103; found: 525.4118. Mp: 178-180°C

**3-*O*-isobutyrylursolic acid (2f)**

The compound **2f** was prepared using isobutyric anhydride. Yellow powder, yield = 45%. IR (ATR, cm^-1^): 2934 (OH acid); 2872 (C-H); 1731(C=O ester); 1692 (C=O acid); 1455 (C-O). ^1^H NMR (300 MHz, CDCl_3_ ), δ (ppm): 0.79 (t, H, CH-5); 0.79 (s, 3H, CH_3_-25); 0.79 (d, 3H, CH_3_-30); 0.79 (s, 3H, CH_3_-24); 0.88 (m, H, CH-20); 0.88 (s, 3H, CH_3_-26); 0.88 (d, 3H, CH_3_-29); 0.97 (t, 2H, CH_2_-11); 0.97 (s, 3H, CH_3_-23); 1.09 (s, 3H, CH_3_-27); 1.15 (s, 3H, CH_3_-33 and 34); 1.20 (m, 2H, CH_2_-7); 1.36 (m, 2H, CH_2_-2 and CH-19); 1.50 (m, 2H, CH_2_-6); 1.55 (t, 2H, CH_2_-15); 1.59 (t, 2H, CH_2_-1); 1.92 (dd, 2H, CH_2_-22); 2.17 (d, H, CH-18); 4.50 (dd, 1H, CH-3); 5.31 (tl, H, CH-12); 2.51 (m, H, CH-32). ^13^C NMR (75 MHz, CDCl_3_), δ (ppm): 15.5 (C-25); 16.6 (C-26); 16.8 (C-29); 18.1 (C-6); 19.1 (C-33 and C-34); 21.1 (C-30); 22.8 (C-23 and C-24); 23.2 (C-27); 23.5 (C-11); 23.6 (C-2); 24.0 (C-16); 28.0 (C-15); 29.6 (C-32); 30.6 (C-21); 32.8 (C-7); 36.7 (C-22); 36.9 (C-10); 37.8 (C-4); 38.2 (C-1); 38.8 (C-20); 39.0 (C-19); 39.5 (C-8); 41.9 (C-14); 47.5 (C-9); 47.9 (C-17); 52.5 (C-18); 55.3 (C-5); 80.3 (C-3); 125.7 (C-12); 137.9 (C-13); 176.7 (C-31); 183.7 (C-28). HRMS (ESI-MS, m/z); [M-H]^-^ calcd. for C_34_H_54_O_4_: 525.8103; found: 525.4111. Mp: 238-240°C

**3-*O*-succinylursolic acid (2g)**

The compound **2g** was prepared using succinyl anhydride. Yellow powder, yield = 24%. IR (ATR, cm^-1^): 2924 (OH acid); 1708 (C=O ester); 1688 (C=O acid); 1455 (C-O). ^1^H NMR (300 MHz, CDCl_3_ ), δ (ppm): 0.67 (t, H, CH-5); 0.70 (s, 3H, CH_3_-25); 0.76 (s, 3H, CH_3_-24); 0.81 (d, 3H, CH_3_-30); 0.88 (m, H, CH-20); 0.90 (t, 2H, CH_2_-11); 0.97 (s, 3H, CH_3_-23); 1.01 (d, 3H, CH_3_-29); 1.06 (s, 3H, CH_3_-27); 1.24 (m, 2H, CH_2_-7); 1.27 (m, H, CH-19); 1.34 (m, 2H, CH_2_-2); 1.43 (t, 2H, CH_2_-21); 1.46 (m, 2H, CH_2_-6); 1.47 (s, H, CH-9); 1.56 (t, 2H, CH_2_-15); 1.59 (t, 2H, CH_2_-1); 1.81 (t, 2H, CH_2_-16); 1.86 (dd, 2H, CH_2_-22); 2.13 (d, H, CH-18); 2.48 (t, 2H, CH_2_-32); 2.58 (t, 2H, CH_2_-33); 4.49 (dd, H, CH-3); 5.40 (tl, H, CH-12). ^13^C NMR (75 MHz, CDCl_3_), δ (ppm): 15.7 (C-25); 16.9 (C-26 and C-29); 18.3 (C-6); 21.3 (C-30); 22.4 (C-23 and C-24); 23.4 (C-2, C-11 and C-27); 23.8 (C-16); 28.1 (C-15); 28.2 (C-32 and C-33); 30.7 (C-21); 33.0 (C-7); 36.8 (C-10 and C-22); 37.1 (C-4); 37.9 (C-20); 38.4 (C-1 and C-19); 39.0 (C-8); 42.1 (C-14); 47.6 (C-17 and C-9); 52.7 (C-18); 55.5 (C-5); 80.8 (C-3); 125.9 (C-12); 138.1 (C-13); 173.9 (C-31); 180.2 (C-34); 184.5 (C-28). HRMS (ESI-MS, m/z); [M+Na]^+^ calcd. for C_34_H_52_O_6_Na: 579.7879; found: 579.3644. Mp: 200-204°C

**3-*O*-dichloroacetylursolic acid (2h)**

The compound **2h** was prepared using dichloroacetic anhydride. Yellow powder, yield = 60%. IR (ATR, cm^-1^): 2925 (OH acid); 1748 (C=O ester); 1687 (C=O acid); 1460 (C-O); 814 (C-Cl). ^1^H NMR (300 MHz, CDCl_3_ ), δ (ppm): 0.69 (d, H, CH-5); 0.74 (s, 6H, CH_3_-23 and CH_3_-24); 0.79 (d, 3H, CH_3_-30); 0.87 (s, 3H, CH_3_-26); 0.88 (d, 3H, CH_3_-29); 0.92 (t, H, CH-11), 0.92 (m, H, CH-20); 0.94 (s, 3H, CH_3_-25); 1.04 (s, 3H, CH_3_-27); 1.23 (m, 2H, CH_2_-7); 1.27 (m, 3H, CH_2_-2 and CH-19); 1.53 (m, 6H, CH_2_-6, CH_2_-9, CH_2_-21); 1.55 (t, 2H, CH_2_-15); 1.57 (t, 2H, CH_2_-1); 1.83 (t, 2H, CH_2_-16); 1.86 (dd, 2H, CH_2_-22); 2.19 (d, H, CH-18); 4.64 (dd, H, CH-3); 5.25 (tl, H, CH-12); 5.98 (s, H, CH-32). ^13^C NMR (75 MHz, CDCl_3_), δ (ppm): 15.4 (C-25); 16.5 (C-26); 16.8 (C-29); 18.0 (C-6); 21.1 (C-30); 23 (C-23 and C-24); 23.2 (C-27); 23.5 (C-11); 23.9 (C-16); 24.0 (C-2); 27.9 (C-15); 30.5 (C-21); 32.8 (C-7); 36.6 (C-22); 36.8 (C-10); 37.9 (C-4); 38.1 (C-1); 38.8 (C-20); 38.9 (C-19); 39.5 (C-8); 41.9 (C-14); 47.4 (C-9); 48.0 (C-17); 52.5 (C-18); 55.2 (C-5); 64.7 (C-32); 85.0 (C-3); 125.7 (C-12); 137.8 (C-13); 164.3 (C-31); 184.2 (C-28). Anal. Calcd. for C_32_H_48_Cl_2_O_4_: Cl, 12.49; found: Cl 13.17, 240-242°C

**3-*O*-trifluoroacetylursolic acid (2i)**

The compound **2i** was prepared using Bis(trifluoroacetic) anhydride. Yellow powder, yield = 100%. IR (ATR, cm^-1^): 2940 (OH acid); 2879 (C-H); 1775 (C=O ester); 1688 (C=O acid); 1158 (C-F).^1^H NMR (300 MHz, CDCl_3_ ), δ (ppm): 0.75 (t, H, CH-5); 0.78 (s, 3H, CH_3_-24 and CH_3_-25); 0.85 (d, 3H, CH_3_-29 and 30); 0.87 (s, 3H, CH_3_-26); 0.91 (m, H, CH-20); 0.92 (t, 2H, CH_2_-11); 0.95 (s, 3H, CH_3_-23); 1.08 (s, 3H, CH_3_-27); 1.25 (m, 2H, CH_2_-7); 1.33 (m, 2H, CH_2_-2); 1.36 (m, H, CH-19); 1.40 (t, 2H, CH_2_-21); 1.44 (m, 2H, CH_2_-6); 1.50 (s, H, CH-9); 1.54 (t, 2H, CH_2_-1); 1.63 (t, 2H, CH_2_-15); 1.81 (t, 2H, CH_2_-16); 1.85 (dd, 2H, CH_2_-22); 2.17 (d, H, CH-18); 4.7 (dd, 1H, CH-3); 5.24 (tl, H, CH-12). ^13^C NMR (75 MHz, CDCl_3_), δ (ppm): 15.4 (C-25); 16.3 (C-26); 16.8 (C-6 and C-29); 21.0 (C-24, C-23 and C-30); 23.5 (C-2, C-11 and C-16); 23.5 (C-27); 27.8 (C-15); 29.5 (C-7 and C-21); 36.8 (C-10 and C-22); 37.9 (C-4 and C-20); 38.8 (C-1 and C-19); 39.0 (C-8); 41.9 (C-14); 47.4 (C-9); 47.9 (C-17); 52.6 (C-18); 55.2 (C-5); 86.2 (C-3); 106.7 (C-31); 125.5 (C-12); 139.5 (C-13); 157.1 (C-32); 182.3 (C-28). HRMS (ESI-MS, m/z); [M-H+OH]^+^ calcd. for C_32_H_47_F_3_O_4_: 569.7355; found: 569.3632. Mp: 224-226°C

**3-*O*-benzoylursolic acid (2j)**

The compound **2j** was prepared using benzoic anhydride. White powder, yield = 100%. IR (ATR, cm^-1^): 2924 (OH acid); 2859 (C-H); 1690 (C=O acid); 1728 (C=O ester); 1460 (C-O). ^1^H NMR (300 MHz, CDCl_3_ ), δ (ppm): 0.69 (t, H, CH-5); 0.73 (s, 3H, CH_3_-25); 0.75 (s, 3H, CH_3_-24); 0.80 (d, 3H, CH_3_-30); 0.87 (s, 3H, CH_3_-26); 0.89 (m, H, CH-20); 0.92 (s, 3H, CH_3_-23); 0.93 (t, 2H, CH_2_-11); 0.94 (d, 3H, CH_3_-29); 1.04 (s, 3H, CH_3_-27); 1.21 (m, 2H, CH_2_-7); 1.30 (m, H, CH-19); 1.32 (m, 2H, CH_2_-2); 1.44 (t, 2H, CH_2_-21); 1.47 (m, 2H, CH_2_-6); 1.49 (s, H, CH-9); 1.54 (t, 2H, CH_2_-1); 1.62 (t, 2H, CH_2_-15); 1.81 (t, 2H, CH_2_-16); 1.86 (dd, 2H, CH_2_-22); 2.13 (d, H, CH-18); 4.70 (dd, 1H, CH-3); 5.32 (tl, H, CH-12); 7.98 (m, H, CH-33 and 37); 7.98 (m, H, CH-34; 35; 36 and 37). ^13^C NMR (75 MHz, CDCl_3_), δ (ppm): 15.4 (C-25); 16.8 (C-26 and C-29); 18.2 (C-6); 21.0 (C-30); 22.6 (C-23 and C-24); 23.4 (C-11 and C-27); 23.6 (C-2 and C-16); 27.9 (C-15); 30.5 (C-21); 32.7 (C-7); 36.7 (C-10 and C-22); 37.0 (C-4); 38.4 (C-1 and C-20); 38.8 (C-19); 39.1 (C-8); 41.9 (C-14); 47.5 (C-9 and C-17); 52.5 (C-18); 55.4 (C-5); 81.6 (C-3); 125.7 (C-12); 128.6 (C-34 and C-36); 129.5 (C-37); 129.8 (C-33); 130.1 (C-32); 132.8 (C-35); 137.8 (C-13); 166.2 (C-31); 184.4 (C-28). HRMS (ESI-MS, m/z); [M+Na]^+^ calcd. for C_37_H_52_O_4_Na: 583.8179; found: 583.5770. Mp: 218-220ºC
